# “Scan Me!”: a rehabilitation approach at the intersection between digital interventions and mountain-therapy

**DOI:** 10.1192/j.eurpsy.2024.474

**Published:** 2024-08-27

**Authors:** A. Barbieri

**Affiliations:** Department of Mental Health, ASL CN1, Cuneo, Italy

## Abstract

**Introduction:**

Mountain-therapy is a therapeutic-rehabilitative approach aimed at secondary prevention, treatment, and rehabilitation of individuals with different pathologies or disabilities. Interventions in this field are based on potentially transformative dimensions of the mountain environment. Activities can include trekking, climbing, hiking, speleology, and winter sports. Benefits associated with these interventions are related to physical health as well as to rehabilitation in the domain of mental health and to the promotion of healthier lifestyles.

**Objectives:**

The pilot project named “Scan Me!” has been developed by mental health services (*Centro Diurno*) of Cuneo (Italy), drawing on their long-standing experience with Mountain-therapy. The aim was to improve the efficacy of mountain-based activities, introducing elements of digitalisation able to actively engage service users and the broader community.

**Methods:**

“Scan Me!” introduces an innovative activity of mapping, communicating, and digitising the mountain environment. The intervention includes: i) participatory identification of thematic areas (e.g. history of a place; local biodiversity; ancient practices); ii) exploration of the identified areas through readings, interviews and research; iii) preparing of messages (texts, pictures, videos) that the group wishes to convey; iv) creation of QR-codes containing the messages; v) positioning of QR-codes along mountain trails during dedicated excursions; vi) setting up of online surveys to get feedback from QR-codes’ users; vii) group discussion of feedbacks and the overall experience. The project includes monitoring and evaluation tools, such as activity forms (filled in with observational data by mental health professionals), self-administered questionnaires for participants, and engagement indicators.

**Results:**

Findings show that the project enhances the therapeutic-rehabilitative value of mountain-based activities, such as increased self-esteem and self-efficacy that follow the completion of a route and relational skills developed within a group. The project shows encouraging results in the planning ability area (identification of themes, setting up of messages, creation and positioning of QR-codes). Being rooted in participants’ interests, the project promotes service users’ knowledge, its sharing with the group and with the general public (mountain visitors). Furthermore, the project implies group reflection, commitment to a concrete objective, and attunement with the recipients of messages (which needed to be tailored for heterogeneous audiences – e.g.: hikers, students, tourists). Lastly, the project is youth-friendly, allowing services to engage a group they aim, but often struggle, to reach.

**Conclusions:**

The pilot encourages further research to understand the potential of rehabilitation tools at the intersection between nature-based and digital mental health interventions.

**Disclosure of Interest:**

None Declared

